# Implant‐bone‐interface: Reviewing the impact of titanium surface modifications on osteogenic processes in vitro and in vivo

**DOI:** 10.1002/btm2.10239

**Published:** 2021-07-12

**Authors:** Theresia Stich, Francisca Alagboso, Tomáš Křenek, Tomáš Kovářík, Volker Alt, Denitsa Docheva

**Affiliations:** ^1^ Experimental Trauma Surgery, Department of Trauma Surgery University Regensburg Medical Centre Regensburg Germany; ^2^ New Technologies Research Centre University of West Bohemia Pilsen Czech Republic; ^3^ Clinic and Polyclinic for Trauma Surgery, University Regensburg Medical Centre Regensburg Germany

**Keywords:** bone‐implant‐interface, in vivo and in vitro, osteogenic differentiation, osteointegration, surface modifications, titanium implants

## Abstract

Titanium is commonly and successfully used in dental and orthopedic implants. However, patients still have to face the risk of implant failure due to various reasons, such as implant loosening or infection. The risk of implant loosening can be countered by optimizing the osteointegration capacity of implant materials. Implant surface modifications for structuring, roughening and biological activation in favor for osteogenic differentiation have been vastly studied. A key factor for a successful stable long‐term integration is the initial cellular response to the implant material. Hence, cell–material interactions, which are dependent on the surface parameters, need to be considered in the implant design. Therefore, this review starts with an introduction to the basics of cell–material interactions as well as common surface modification techniques. Afterwards, recent research on the impact of osteogenic processes in vitro and vivo provoked by various surface modifications is reviewed and discussed, in order to give an update on currently applied and developing implant modification techniques for enhancing osteointegration.

**Abbreviations**
(B)MSCs: (bone marrow derived) mesenchymal stem cells
BIC: bone implant contact
CHAp: carbonated hydroxyapatite
ECM: extracellular matrix
H: height
HAp: hydroxyapatite (coating)
MA: machined (implant surface)
MAO: micro‐arc oxidation
Ø: diameter
*R*_a_
: roughness average
RTV: removal torque value
Th: thickness
Ti: titanium
TiN_*x*_O: titanium‐nitride‐oxide
TiO_2_
: titanium dioxide, titania

## 1 FOREWORD AND REVIEW SCOPE

Titanium (Ti)—commercially pure titanium and its alloys, usually grade 5 Ti6Al4V—are commonly used for dental and orthopedic implant applications due to their excellent resistance to corrosion, biocompatibility properties, mechanical strength and elastic modulus, which is closer to bone compared to other metals.^1^, ^2^, ^3^ As bones have a major functional importance including structural composition of the skeleton, load bearing, and motion support of the human body, a skeletal impairment or disease greatly affects the quality of life of a patient.^4^ Therefore, it is of great importance to maintain bone function throughout life and in the case of terminal disease stage or severe injury, bone replacement by implants is the primary choice for treatment. Dental implants composed of titanium are widely used and show excellent long‐term results. In orthopedics, titanium is used for uncemented implants, which are in direct contact to the bone tissue. Cementless fixation requires bone tissue to attach to the implant surface to secure the integration of the implant. For that reason, cementless implants are primarily used for bones of good tissue quality, such as in healthy young patients, and are not suitable for bones with lower mineral density, such as in aged and osteoporotic patients. However, developing an implant that allows cementless fixation also in compromised bone would offer clear benefits, such as protection of native bone tissue and avoidance of incorporation of body foreign substances (bone cement). In addition, bone implants, despite the fact that they are well established, still face the problem of implant failure due to two leading reasons—implant loosening owing to insufficient bone integration and/or the production of fibrous tissue or infection. Therefore, there is a continuous scientific effort toward the development of innovative implant materials (surfaces) that can (i) stimulate healing and enhance osteointegration, independently of the bone quality, (ii) act inhibitory for infections, and (iii) prolong the longevity of an implant.^5^ Osteointegration arises from the physical and chemical interaction between the implant surface and the bone tissue.^6^, ^7^, ^8^ Evaluating the biological responses triggered by surface modifications can be used to guide the cellular response at the bone implant interface for achieving implant surfaces with augmented osteointegration.^6^, ^7^, ^8^ Thus, nature‐inspired implant surfaces that are very similar to the native bone tissue topography at the macro‐ and nano‐scale as well as that can be further functionalized to simulate the bone biochemical milieu are of great interest to the field.^9^, ^10^, ^11^

In this review, we start with a foreword on titanium implants and the review scope, followed by a synopsis on the discrete interactions between cells and biomaterials and an overview of surface modifications enhancing osteogenic differentiation. Next, literature on recent research regarding implant surface modifications and their impact on osteogenic processes in vitro and in vivo is discussed in detail.

Surface modifications for improved implant performance is a vastly studied area. We were particularly interested to obtain the latest information of research, focusing on biological assessment of implant surface modification techniques with the overall aim to enhance osteointegration. Literature search was conducted via the National Center for Biotechnology Information (NCBI) database. For the informational chapters 1–3 and Table 1, articles (approximately 50, many of them review articles) dealing with general information on titanium implants and types of surface modifications and cell to material interactions and integrin signaling were selected. For chapters 4 and 5, plus Tables 2 and 3, a NCBI databank search was performed as follows: (1) the keywords, titanium, titanium alloys, osteogenesis, osseointegration, biomaterials and combinations of these keywords were used; (2) filters were set for publication date within the past 5 years and English language; and (3) articles were excluded if there were duplicates, abstract only and no accessibility to full text. The articles (approximately 200) were then thoroughly screened for data containing cellular response on osteogenic differentiation in vitro and osteointegration in vivo, resulting in the analysis of approximately 50 research papers for this review.

## 2 DISCRETE INTERACTIONS BETWEEN CELLS AND MATERIAL SURFACES

**FIGURE 1:**
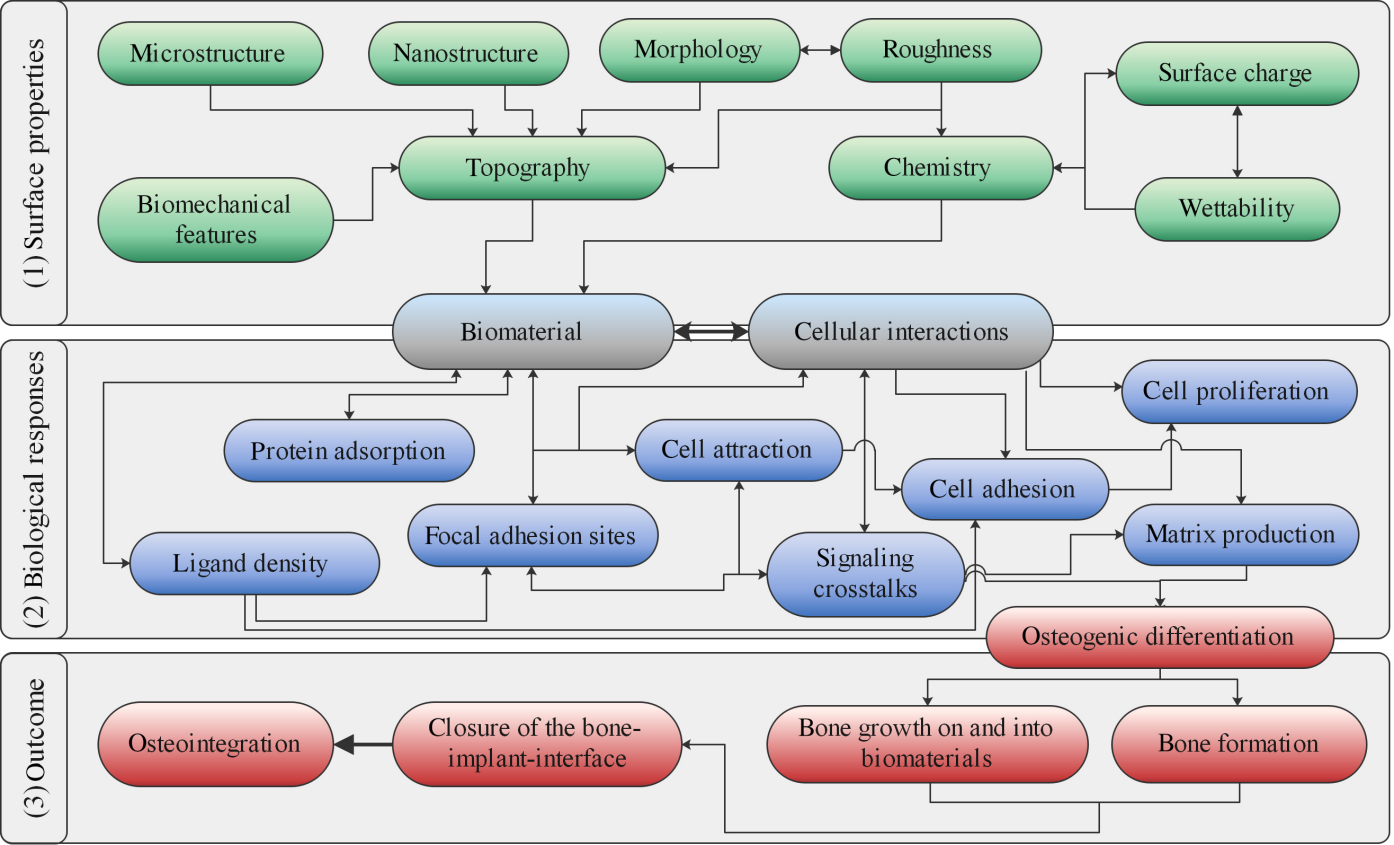
Visualization of the interrelation of biomaterial properties and the biological (osteogenic) response. The interrelationship of surface characteristics of a biomaterial and the cell response is a complex mechanism dependent on numerous factors that are accountable for successful osteointegration. (1) Various surface properties, ranging from topographical to chemical features, affect (2) the biological and cellular response to biomaterials (e.g., ligand density, protein adsorption, cell adhesion, cell signaling) and finally (3) determine the biological outcome of an implant (surface) in terms of osteogenic differentiation and osteointegration

The surface of an implant is in direct contact with the host tissue, for example, bone tissue. Therefore, the surface properties are a main determining factor for the subsequent complex cell behavior at the bone‐implant interface in vivo as well as for the cell response in vitro (Figure 1).^12^ Different parameters, for instance surface topography, chemistry, charge and culture conditions (in vitro) or physiological environment (in vivo), impact the discrete interactions between cells and the biomaterial.

Interestingly, the same basic substrate can provoke different cell responses when exhibiting different nanostructures, leading, for example, to modulations in cell adhesion, motility and signaling pathways.^13^ It is important to understand the dynamic interactions between biomaterials and adhering cells, as this affects cell proliferation, differentiation, migration and consequently, the integration of the biomaterial into the host tissue.^14^

Bone tissue has a mineralized macroporous structure with nano‐scale components that determine its strength. Inorganic hydroxyapatite (HAp) constitutes the major part of the mineralized component. The organic extracellular matrix (ECM) predominantly consists of collagen type I and the bone cells—osteogenic progenitor cells, osteoblasts, osteocytes, and osteoclasts. Naturally, the hierarchical structure of the bone (from nanolevel, e.g., collagen molecules, minerals, to microlevel, e.g., the osteon) guides the bone cells in their tissue specific behavior.^13^, ^15^ Thus, titanium implant surfaces should ideally have characteristics similar to the native bone topography in order to facilitate the desired cell responses which in turn enable osteointegration.^10^, ^13^, ^15^ In this manner, it may be possible that even aged and osteoporotic cells could be stimulated and have an enhanced osteogenic differentiation potential.

**FIGURE 2:**
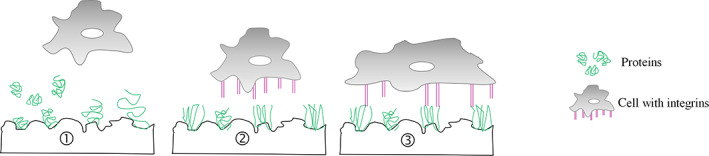
Cartoon depicting the cell receptor recognition of biomaterials. The initial response of cells to biomaterials occurs via surface receptors, such as integrins. (1) First, water, other solubles of the biofluid (not depicted), and proteins (depicted in green) attach to the implant surface and (2) adopt a certain conformation depending on the surface properties. (3) Cells are able to sense and attach to the proteins, and form focal adhesions on the surface. *Source*: Adapted from Kim et al.^16^

After an implant or biomaterial is exposed to biofluids, the adsorption of water, serum molecules, proteins and cells (Figure 2, step 1) is determined by the physicochemical state of the surface, mainly its chemistry and charge. ^16^ Following their adsorption to the surface, proteins adapt to a specific conformation, which depends upon the surface properties. The initial cell linkage to the material is governed by composition, density and conformation of the adsorbed proteins. Subsequently, cells close to the surface start filopodial sensing via integrins (Figure 2, step 2). Integrins are glycoprotein cell surface receptors that interact with ECM adhesive proteins, they cluster in the so‐called focal adhesion points and are thereby involved in cell attachment to biomaterials. Cellular integrins bind to formed focal adhesions, forces are transmitted via the cell membrane and a downstream filament cascade, resulting in rearrangement of cellular cytoskeleton (Figure 2, step 3). Interactions between integrins and ECM proteins occurs via recognition of amino acid sequence domains (e.g., RGD (Arg Gly Asp)) that is found in fibronectin, osteoprotegerin and bone sialoprotein, or GFOGER (glycine‐phenylalanine‐hydroxyproline‐glycine‐glutamate‐arginine) for collagen type I. The impact of surface characteristics on cell morphology and differentiation is mediated via integrins, as surface properties interfere with integrins and influence interactions between integrins and their ligands. The integrin signaling cross‐talks with signaling pathways of growth factors, guiding the behavioral pattern of MSCs and bone cells.^12^, ^14^, ^17^, ^18^ For example, fibronectin, an adhesive protein considered as pro‐osteogenic, interacts with cells via integrin focal adhesion points. Thereby, it is controlling cell activity and promotes osteogenic differentiation of MSCs. Osteoblasts were shown to attach to 2D surfaces in vitro via integrins, whereby the focal adhesion site formation relied on the integrin activation state.^12^, ^14^, ^19^, ^20^, ^21^

**FIGURE 3:**
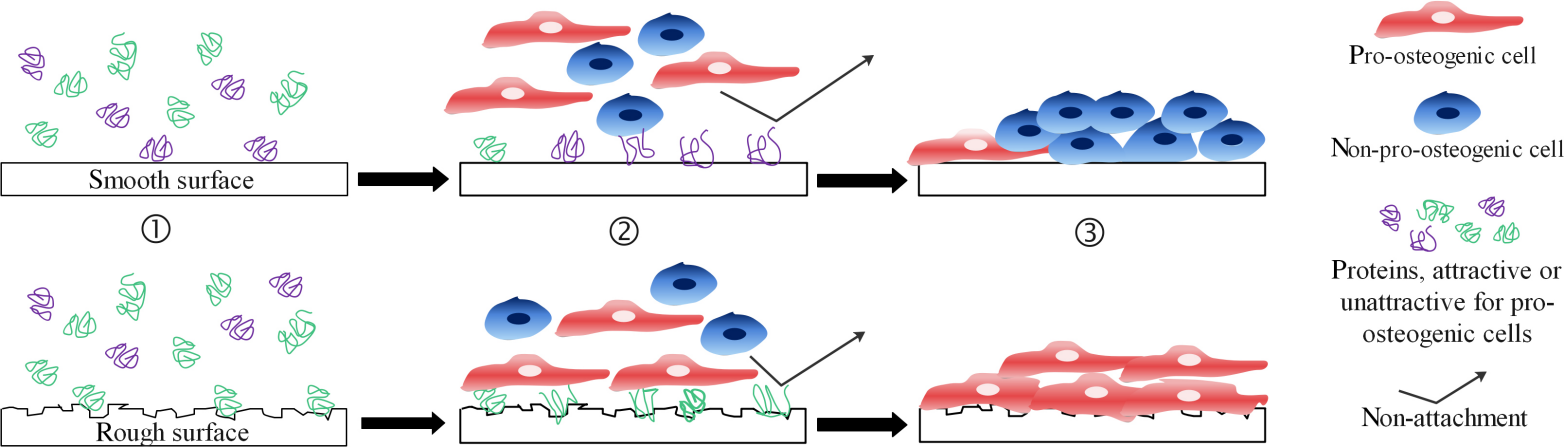
Cartoon showing the basic cell to material interactions on smooth or textured rough surfaces. (1) The surface structure and roughness provoke a different protein adsorption. (2) This protein pattern affects cell attraction and attachment and (3) cell proliferation and following differentiation and maturation. *Source*: Adapted from Khullar et al.^22^

Biomaterials devoid of surface roughness in the micro‐ and nano‐scale range have shown to hinder cell osteogenic differentiation. Rougher surfaces (mean average roughness *R*_a_ > 0.5 μm) were correlated to increased bone to implant contact (BIC) and described to be preferred by bone cells compared to smooth surfaces. Figure 3 graphically depicts major differences between smooth and roughened surfaces. On smooth surfaces, less pro‐osteogenic but rather fibrotic cells attach and proliferate, which can result in fibrous tissue formation and implant loosening in vivo.^22^ However, such surfaces have been shown to achieve sufficient osteointegration in dentistry.^23^ In general, pro‐osteogenic cells are more favorable to attach, proliferate and differentiate on rough nano‐patterned surfaces, thereby reducing the risk of undesirable fibrosis.

Cells exposed to roughened biomaterials exhibit more focal contact points, cell adhesion and increased proliferation. These differences in cell response also rely on the integrin reaction to the surface topography, which is determined by the structure (roughness, size, morphology) and the mechanical properties (stiffness, deformity, rigidity, elasticity). Integrins, plasma membrane receptors, can sense the biomechanical niche and initiate biochemical signaling cascades regulating cell behavior.^24^, ^25^, ^26^ The exact degree of nano‐scale influence on the cell response, however, depends on the cell type.^13^, ^22^ The biomaterial nano‐scale features can enrich protein adsorption and modulate the arrangement of the cytoskeleton (Figure 2, step 3) leading to an improved osteogenic stimulation of cells.^16^, ^27^ For example, osteoblasts exhibit an enhanced collagen production and calcification processes when cultured on rough surfaces.^28^, ^29^ It has also been shown that the combination of multiple length‐scale features of the implant topography correlates with increased osteoblast differentiation.^30^

**FIGURE 4:**
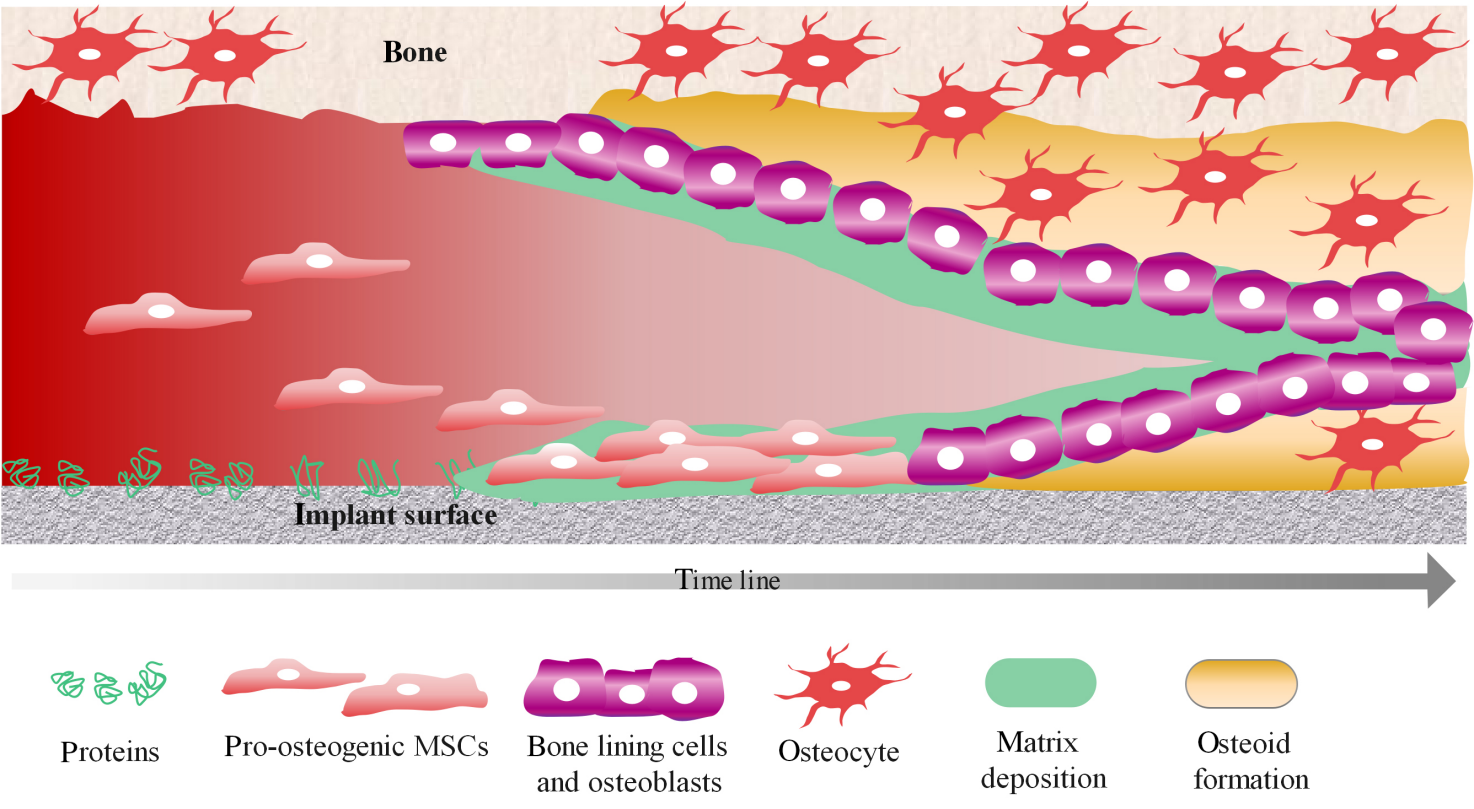
Simplified graphical overview of the cell response at the bone implant interface in terms of osteogenic differentiation. At first, water, serum molecules and proteins are adsorbed to the implant surface and cells are thereby attracted to the implant site. This is followed by cell attachment, their subsequent differentiation toward osteoblastic cells and matrix deposition; thus, ending with the final process of osteoid maturation, osteocyte differentiation and the closure of the gap between bone and the implant material. *Source*: Inspired by Puleo et al.^109^

Biomaterials incorporated in the bone tissue form the so‐called bone‐implant interface at the implant site. Figure 4 schematically shows the cell reaction in terms of osteogenesis and de novo osteoid formation at the interface. After protein adsorption to the implant surface, MSCs are attracted; they attach and start to proliferate. Ideally, due to different biochemical and biomechanical stimuli and the adsorption of serum proteins and growth factors, osteogenic lineage differentiation toward osteoblasts is initiated. Mature osteoblasts secrete matrix, the direct pericellular niche that is rich of collagen I, which incorporates the osteocytes and evolves to form new bone matrix via calcification and mineralization. The composition of the filled bone‐implant interface of successfully integrated implants is similar to the natural bone. Also, the osteocytes of the neighboring native bone tissue can maintain their normal morphology, regardless of the distance to the implant surface, and can even reach toward the implant site with their cellular protrusions.^15^, ^31^

Taken together, biomaterials and their surface properties influence cell behavior. The processes of cell–material interaction along with bone healing around an implant displays complex interactions between the material, different cell types and signaling pathways.^14^, ^18^ It is essential to be conscious about these processes when designing an implant surface. Understanding the discrete cell responses can help modulating the surface features in order to steer the cell toward the desirable biological response.

## 3 IMPLANT SURFACE MODIFICATION TECHNIQUES

This chapter provides a short synopsis on surface modification techniques, for detailed reviews on methodologies, please refer to other reviews, for example, Refs. 32, 33, 34, 35 ^.^

Combined effects of the surface chemistry, topography and the resulting surface energy play essential roles, especially during the early phases of the biological response, and influence the subsequent osteointegration of the implant.^36^, ^37^

The surface properties of a metallic implant material are essentially characterized by its inherent chemical composition and the surface's physical and or biochemical modification(s).^38^ As mentioned above, the topography describes the biomechanical and structural characteristics of the surface. In general, the roughness of a surface on the micro‐scale has been classified into smooth (average roughness *R*_a_ < 0.5 μm), machined/minimal (*R*_a_ = 0.5–1 μm), moderate (*R*_a_ = 1–2 μm) and rough (*R*_a_ > 2 μm).^39^, ^40^

Overall, surface modifications increasing hydrophilicity and roughness exert positive effects on osteogenic differentiation of cells and enhance osteointegration of implants.^41^, ^42^ Hydrophilic and roughened surfaces support cell attachment while roughness at the macro‐ and micrometer scale improve mechanical anchorage of the implant in the bone tissue.^43^, ^44^ Roughening produces an enlarged surface area leading to a broader territory for cell adhesion, bone‐implant‐contact and thus better biomechanical integrity after the bone‐implant‐interface is filled with new bone matrix.^12^, ^45^ Furthermore, surface roughness modifications can also lead to a surface chemistry favorable for osteogenic stimulation. Surface modifications via structural changes influence the physicochemical properties and vice versa, coating with various molecules can affect surface roughness and structure.

**FIGURE 5:**
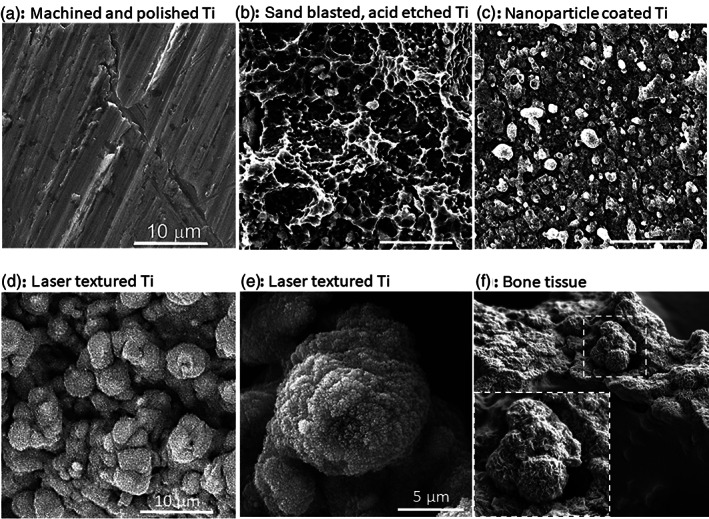
Scanning electron microscope (SEM) images showing examples of titanium surfaces after processing with different surfaces modification techniques. (a) Mechanical polishing, often used as a control in research studies. (b) Sandblasting and acid etching. (c) Pulsed laser deposition of particles. (d and e) Laser texturing by nano‐second pulsed laser. (f) SEM image of bone tissue. Scale bar (a)–(d): 10 μm; scale bar (e): 5 μm; magnification (f): 4000×. *Source*: Representative images (a), (b), (c), (d) and (e) were provided by co‐authors T. Křenek and T. Kovářík; copyright for image (f) was purchased from Science Photo Library/Science Source/Nano Creative. A higher magnification image of the representative image in (a) appears also in the publication Křenek et al. *Surfaces and Interfaces*. 2021;26:101304, https://doi.org/10.1016/j.surfin.2021.101304

Creating a suitable porous and rough morphology is the first step in the development process of a bone implant surface.^9^, ^10^, ^30^ Some commonly used techniques for implant surface roughening are shown in Table 1. For the generation of the basic implant surface roughness, physical (e.g., grinding or laser texturing) and chemical (e.g., acid etching) modification techniques are applied. Figure 5 exemplarily shows titanium surfaces modified with different techniques. Chemical modification techniques, such as acid etching, are more likely to alter the chemical surface composition than physical methods. For example, acid etching of titanium with HCl and H_2_SO_4_ was shown to lead to hydrogen adsorption and formation of stable titanium hydride on the surface.^46^, ^47^ Interestingly, titanium surfaces roughened with physical methods often demonstrate the formation of the so‐called TiO_2_ passivation layer.^48^, ^49^, ^50^ In addition to appropriate macro‐ and micro‐features of an implant, nano‐patterning has been reckoned to play a crucial role for the biological response.^9^, ^27^, ^51^ Despite that the sand blasting and acid etching (SLA)‐treated implants are commonly used in clinics, there are indications that laser texturing provides a more suitable nano‐topography compared to the rather sharp‐edged morphology after SLA treatment. Comparing a scanning electron microscope (SEM) image of a laser textured surface to a SEM image of bone tissue surface, shows their great resemblance (Figure 5). Laser texturing is one of the latest and promising technologies for metal implant surface structuring that allows to design a desired, controlled and reproducible surface geometry at different length‐scales.^52^, ^53^, ^54^ During the manufacturing process, no additional chemicals, which might be harmful, are incorporated into the surface layer. Moreover, in a stochastic manner, laser texturing automatically creates metal nanodroplets on the implant surface, thereby generating a nano‐roughened topography with a foamy, roundly shaped nano‐features.^51^, ^53^, ^54^

**TABLE 1:** Examples of surface modification techniques and coatings for improving surface osteosupportive properties.

Surface roughening and texturing techniques	Coating techniques	Coating substances
Mechanical polishing	Pulsed laser deposition	CaTiO_3_
Blasting	Electrochemical oxidation	Hydroxyapatite (calcium phosphate)
Grinding	Precipitation	Calcium, magnesium, sodium, strontium
Polishing	(Plasma) spraying	Ions with antibacterial properties
Laser texturing	Chemical vaporing	For example, Zr, Cu, Ag
Micro‐arc oxidation	Immersion	Biopolymers
Sonochemical treatment	Sol–gel synthesis	For example, polysaccharides, proteoglycans
Magnetron sputtering	Magnetron sputtering	Proteins (bone related)
Ultraviolet radiation	Alkali treatment	For example, collagen, fibronectin, osteopontin, bone sialo protein
Electron beam physical vapor deposition		Peptides, e.g. RGD
Hydrothermal treatment		
Selective laser melting		

To further enhance the bioactivity of a titanium implant surface, additional ion and molecular functionalization (Table 1) can be carried out with the goals of (1) eliminating proteins which would lead to attachment of unspecific cells, resulting in fibrotic tissue formation or bacterial adhesion; (2) boosting the adherence of desired cell types, that is, osteogenic progenitor cells and osteoblasts; (3) guiding responses of immune cells modulating inflammation during the process of bone healing.^12^ The functionalization is based on the incorporation or binding of inorganic ions or molecules (e.g., magnesium (Mg), calcium (Ca) and strontium (Sr)) and organic molecules (e.g., peptides, proteins and drugs).^11^, ^55^, ^56^ HAp has been investigated as a coating substance for a long time and is still frequently chosen. Its deposition can promote better BIC and bone formation, and is already in clinical use.^57^, ^58^, ^59^, ^60^ The deposition of coating molecules is performed with various methods including plasma spraying, electrochemical/micro‐arc/anodic oxidation, immersion, acid etching and laser ablation (Table 1). Either, molecules are formed automatically but uncontrolled on the surface (indirect coating, e.g., anodic oxidation or immersion); or the molecules are directly deposited on the surface in a controlled density (e.g., plasma spraying, laser ablation).

There has been an enormous advancement in new methods for texturing and biofunctionalizing implants. However, to estimate the translational power of novel surface modifications, thoughtful assessment of the complex cellular and tissue responses is required. Therefore, the following chapters will focus on the output of surface modification techniques on osteogenic processes in vitro and in vivo.

## 4 IMPACT OF SURFACE PROPERTIES ON OSTEOGENIC PROCESSES IN VITRO

Studies to analyze the effect of different titanium implant surface modifications on in vitro osteogenesis were conducted using various mammalian cell lines, such as MSCs or murine calvarial (pre)osteoblasts.^48^, ^61^, ^62^, ^63^, ^64^, ^65^, ^66^, ^67^, ^68^ As summarized in Table 2, the majority of the reviewed studies used disc^48^, ^61^, ^62^, ^63^, ^64^, ^65^, ^69^, ^70^, ^71^ or rectangular^66^, ^67^, ^72^ shaped titanium implants with varying surface modifications, for example, grit‐blasting, magnetron sputtering or acidic treatment.

**TABLE 2:** Overview of surface modifications and their effect on osteogenic differentiation in vitro

Surface properties	Surface modification method	Control surface	Experimental parameters	Time points	Conclusions	Reference
Rough TiO_2_ (*R*_a_ = 10.57 μm)	Grit‐grinding, pulsed (Yb:YAG) laser ablation	Polished TiO_2_	Disc A = 175 mm^2^ Th = 2 mm murine calvarial osteoblast	Day 1, 3, 7, 14	Roughened TiO_2_ surface promoted morphological changes and increased osteoblast differentiation as well as mineralized matrix formation	Mariscal‐Muñoz et al.^48^
Periodic micron/nano‐groove topography (*S*_a_ = 246 nm)	Mirror‐polishing femtosecond (fs) laser irradiation	Mirror‐polished TiO_2_ (*S*_a_ = 32 nm)	L = 10 mm B = 10 mm MC3T3‐E1	Day 21	Fs laser modified TiO_2_ surface promoted osteogenic differentiation and matrix calcification shown by higher gene expression of osteocalcin and osteopontin	Chen et al.^66^
Nano‐porous TiO_2_ pore Ø = 20 nm (*R*_a_ = 9.2 nm) Crystalline phosphate‐containing microstructure TiO_2_ (*R*_a_ = 1.2 μm)	Three‐stage polishing and oxidative nano‐patterning via acid etching	Polished TiO_2_	Disc Ø = 12 mm Th = 2 mm MC3T3‐E1	Days 1, 2, 3	Nano‐porous titania surface affected the cellular biomechanical strength via the formation of cell‐protrusions, abundant filopodia, and increased focal adhesion points	Bello et al.^63^
Disordered mesoporous nanostructured titania (TMS) *R*_a_ > 20 nm Ordered nano‐tubular nanostructured titania (TNT) *R*_a_ > 20 nm	Electron beam physical vapor deposition, sonochemical‐treatment and electrochemical oxidation	Glass *R*_a_ < 5 nm	Th = 400 nm MC3T3‐E1	Hours 3, 24	Cells response differed between the ordered TNT and disordered TMS nanostructured surfaces. TMS surface was more favorable for cell adhesion and proliferation due to increased focal adhesion points	Zhukova et al.^67^
TiN_*x*_O_*y*_‐coated TiO_2_ micro‐roughened surface	Sand blasting and acid etching (SLA) Reactive direct current magnetron sputtering for TiN_*x*_O_*y*_ coating	Micro‐rough TiO_2_	L = 11 mm B = 11 mm H = 0.635 mm HOS cells EA.hy926 cells	Days 3, 7, 14, 21	TiN_*x*_O_*y*_ coating enhance osteoblasts adhesion, spreading, proliferation, and neovascularization of endothelial cells	Moussa et al.^72^
Microporous TiO_2_ containing‐Sr Microporous TiO_2_ containing‐Sr/Ag 0.40 Microporous TiO_2_ containing‐Sr/Ag 0.83 TiN_*x*_O_*y*_‐coated TiO_2_ micro‐roughened surface	Magnetron sputtering with micro‐arc oxidation	Microporous TiO_2_	Wafers Ø = 14 mm Th = 2 mm MC3T3‐E1	Days 1, 7, 14, 21, 28	Microporous TiO_2_ surface containing optimal proportion of Sr/Ag favored osteoblast adhesion and differentiation with sustained antibacterial activity	He et al.^68^
Crystalline phosphate‐containing microstructure TiO_2_ (*R*_a_ = 1.2 μm)	Grit‐blasting using absorbable blast media and hydrothermal treatment in phosphoric acid	Micro‐rough TiO_2_ (*R*_a_ = 1.42 μm)	Disc Ø = 15 mm Th = 2 mm Murine BMSCs, human adipose‐derived MSCs	Week 38	The hydrophilic phosphate ion surface enhanced early cellular functions and osteogenic differentiation	Kwon and Park^64^
Nanorod CHAp Hybrid micro‐/nanorod CHAp Micro‐rod CHAp	Hydrothermal dip coating using hydroxyapatite (HAp) and carbonated hydroxyapatite (CHAp)	Micro/submicron hybrid HAp rods	Disc Ø = 8 mm Th = 1 mm Rat BMSCs	Day 1, 7, 21	CHAp treated surfaces especially the micron–nano‐hybrid surface enhanced cellular adhesion, proliferation, and osteogenic differentiation	Li et al.^73^
TiO_2_ coated with apatite by flame spraying (FS) TiO_2_ coated with apatite by blasting (BC)	Apatite coating by flame spraying and blast coating	Machined surface	Disc Ø = 30 mm Th = 3 mm Human osteoblast‐like cells (Saos‐2)	Day 1, 5, 10, 15	BC surface promoted cell adhesion and proliferation via higher expression of Fibronectin and E‐cadherin, and improved osteogenic differentiation via increased cellular ALP (Alkaline phosphatase) activity	Umeda et al.^71^
TiO_2_ nano‐network structure UV‐treated TiO_2_ nano‐network structure	Mechanical polishing Alkali and high‐intensity ultraviolet treatment	Polished surface	Disc Ø = 15 mm Th = 1 mm Rat BMSCs	Day 1, 3, 7, 14, 21, 28	UV treated surface promoted antibacterial activity and enhanced protein adsorption, cellular adhesion, proliferation and differentiation	Zhang et al.^65^

The appropriate selection and combination of surface modification techniques affects its cellular biocompatibility and influence. For example, an apatite coated titanium dioxide (TiO_2_, titania) surface produced by blasting, performed better in terms of cellular adhesion and proliferation than an apatite coated TiO_2_ surface fabricated by flame spraying.^71^ Moreover, the blasting method achieved increased cellular alkaline phosphatase activity and expression of essential cell–cell and cell–matrix adhesion proteins (e.g., fibronectin and E‐cadherin), indicating enhanced osteogenic ability.^71^

Mariscal‐Munoz et al. found that the micro‐to‐nano surface roughness generated by laser ablation, augmented osteoblast differentiation and matrix mineralization, alongside an increased expression of bone specific genes.^48^ Chen et al. reported enhanced osteogenic differentiation and matrix calcification of mouse pre‐osteoblasts cultured on a TiO_2_ micro–nano‐grooved pattern, fabricated via femtosecond laser irradiation.^66^ The enhanced roughness of this TiO_2_ surface positively affected the surface energy, which primarily governs initial protein and cell adhesion and the subsequent induction of cell differentiation and ECM maturation.

Bello et al. showed that a nano‐porous TiO_2_ surface produced via oxidative chemical treatment promoted the formation of cellular protrusions and increased focal adhesion processes, shown by a significantly higher expression of genes associated with cell matrix sensing and adhesion.^63^ Different adhesion and migratory patterns were observed in pre‐osteoblasts cultured on ordered (nano‐tubular) or disordered (mesoporous) titanium nanotopographies.^67^ Cells cultivated on ordered nanotubes developed an elongated polarized morphology with decreased focal adhesion. In contrast, the disordered mesoporous surface exhibited polygonal shaped cells with more focal adhesions and enhanced cell proliferation.

The positive biological influence of the implant surface topography and chemistry is also evident at the micro‐scale and depends on the combination of micro‐ and nano‐scale features. A study by Moussa et al. demonstrated that the titanium‐nitride‐oxide (TiN_*x*_O_*y*_) coating of a micro‐rough titanium surface had a synergistic effect on the initial spreading and adhesion of osteoblasts in comparison to the standard micro‐rough TiO_2_ surface.^72^ The TiN_*x*_O_*y*_ coating enabled augmented osteoblast adhesion, spreading and proliferation on collagen via the integrin binding α1β1 or α2β1 in association with. Moreover, this coating also exerted positive effects on endothelial and immune cells.

An interesting bioactive effect was also observed for incorporated strontium Sr particles in roughened microporous scaffold. Specifically, this combination significantly improved osteoblast spreading and differentiation.^68^ A similar effect was reported by Kwon et al. for crystalline phosphate incorporated into a grit‐blasted micro‐rough titanium implant.^64^ This surface exhibited a long‐term superhydrophilic effect that promoted cell adhesion, spreading, proliferation and early osteogenic differentiation of multipotent murine, as well as human MSCs. A recent study by Li et al. showed enhanced cellular response toward titanium surfaces coated with highly carbonated hydroxyapatite (CHAp) in varying concentrations.^73^ The 8% CHAp crystals exhibited nanorod structures, the 12% CHAp crystals produced a hybrid of nano‐ and micro‐rods and the 16% CHAp crystals were mostly micro‐rods. Intriguingly, the biomimetic 12% variant demonstrated the highest hydrophilicity, improved surface wettability, cell adhesion, protein adsorption and osteogenesis, suggesting enhanced physicochemical properties of the micro‐ and nano‐textured combination.

Some of the surface modification techniques had valuable additional effects and led to the achievement of material exerting antibacterial properties. The functionalization of a porous TiO_2_ surface with strontium and an optimal concentration of silver that was applied using a magnetron sputtering technique combined with micro‐arc oxidation, demonstrated strong antibacterial effects for up to 28 days.^68^ Besides, Zhang et al. reported improved osteogenic effects coupled with increased antibacterial activity after exposing alkali‐treated TiO_2_ to high‐intensity ultraviolet radiation.^65^ The ultraviolet treatment created a superhydrophilic environment favoring protein adsorption that positively influenced cellular attachment and proliferation, while preventing the initial attachment and growth of bacteria.

Taken together, the material substrate niche directly influences the initial cell to surface interaction and the resulting cellular processes. Moderately rough and porous nano‐surfaces promoted better cell response than smooth and an irregular surface organization is more favorable for osteogenic lineage differentiation than ordered. Additional UV treatment or coating with certain molecules can positively enhance both the biocompatibility and the antimicrobial activity of a titanium implant surface. It will be of great interest to the field that in future research thorough investigations on the impact of surface modification techniques are performed with larger cohorts of human primary cells (e.g., healthy, osteoporotic bone cells) instead of cell lines. This would lead to obtaining valuable information regarding the osteoinductive capacities of the surface characteristics and further improve the translational power and clinical relevance of such studies.

## 5 IMPACT OF SURFACE PROPERTIES ON OSTEOGENIC PROCESS IN VIVO

In order to truly elucidate the enhancing effect of various novel titanium implant surface modifications on osteointegration, in vivo studies involving direct contact between bone tissue and the implant surface are very crucial. The studies included in this review mainly employed commercially pure titanium implants of various shapes in millimeter scale. Combinations of different surface modification techniques were employed by independent investigators to develop new titanium implant surface topographies for improved osteointegration. The implants were embedded in various anatomical regions of different experimental animal models. The biological effects of the newly designed titanium implant surfaces were compared to standard smooth or rough surfaces at the early and late stages of bone formation. The level of osteointegration was assessed using important histomorphometric parameters such as the BIC which expresses the percentage of new or existing bone connected to the implant surface. For determining the strength of the interaction between bone and the incorporated implant surface, the removal torque value (RTV) of the implant was frequently analyzed. Table 3 gives an overview on the included research articles, the utilized surface modifications and achieved outcome. In the subsequent sections, the included studies are discussed in more detail.

**TABLE 3:** Overview of surface modifications and their effect on osteointegration in vivo

Surface properties	Surface modification method	Control surface	Experimental parameters	Time points	Conclusion	Reference
MAO‐treated TiO_2_ MAO‐treated TiO_2_ layered with Sr	Micro‐arc oxidation (MAO) and electrochemical treatment	Untreated TiO_2_	*L* = 10 mm *B* = 10 mm *H* = 1 mm Canine mandible	Week 6	The MAO‐Sr coating induced faster bone formation and osseointegration than the other two groups	Zhang et al.^74^
Moderately rough micro‐structured TiO_2_ surface	Sandblasting and acid‐etching (SLA)	Machined (MA)TiO_2_ surface	Screw Ø = 1.5 mm *L* = 6.5 mm Rabbit tibia	Week 12	SLA surface showed significantly higher removal torque compared to control. However, both groups showed similar BIC	Maino et al.^49^
Dual acid‐etched micro‐nano‐textured surface	Dual acid‐etching and Nano‐texture blasting	Dual acid‐etched micro‐textured surface	Rectangular plate *L* = 1.3 mm *B* = 2.5 mm *H* = 4 mm Rat distal femur	Day 9	The nanostructured surface conferred greater bone bonding and strength relative to the acid‐etched surface	Coelho et al.^75^
Laser micro‐textured TiO_2_	Pulsed laser texturing	MA TiO_2_	Screws Ø = 3.8 mm *L* = 9 mm Sheep iliac crest	Week 8	Laser treated surface showed superior mechanical strength and BIC compared to the machined surface	Trisi et al.^50^
3D produced rough and irregular surface	Selective laser melting (SLM); machining (MA), anodic oxidation	MA surface with anodic oxidation	Disc Ø = 11.5 mm *H* = 4 mm Canine mandible	Week 9	No significant difference between groups (bone volume, BIC); removal torque values (RTVs) of SLM higher than MA but lower than surface with anodic oxidation treatment	Shaoki et al.^77^
TiO_2_ nanotube TiO_2_ nanotube + rhBMP‐2 TiO_2_ nanotube + Ibuprofen	Anodic oxidation, dip coating	MATiO_2_	Screw Ø = 1,6 mm *L* = 6 mm Rabbit leg	Week 8	BIC of Ibuprofen loaded TiO_2_ was higher than that of rhBMP2 that was higher than unloaded TiO_2_ while the machined was the lowest	Jang et al.^76^
Micro/nano‐hybrid roughened TiO_2_ surface (*S*_a_ = 3.35 μm)	Selective laser ablation	Machined TiO_2_ surface (*S*_a_ = 0.27 μm)	Screw Ø = 3.75 mm *L* = 5 mm Rabbit tibial metaphysis	Week 8	Laser‐treated surface showed superior biomechanical anchorage compared to machined surface	Shah et al.^78^
Porous micro–nanoroughened TiO_2_ surface (*R*_a_ = 2.47 μm)	Grit‐blasting, acid etching and laser sintering	Solid micro–nanorough TiO_2_ surface (*R*_a_ = 2.66 μm)	Rod Ø = 3.8 mm *L* = 8 mm Rabbit femur	Week 10	Porous surface enabled superior bone in‐growth compared to the solid surface	Cohen et al.^79^
Hydrophilic ultra‐fine‐grained nano‐patterned surface ufgTi (max. Grain size 300 nm)	Equal channel angular pressing and SLActive treatment	SLActive	Screws Ø = 4.8 mm *H* = 6 mm Miniature pig maxilla and mandible	Week 4, 8	ufgTi showed superior mechanical property. The hydrophilic surface supported high levels of osteointegration even in compromised bone	Chappuis et al.^80^
Micro–nano‐porous oxidized TiO_2_ surface (*R*_a_ = 1.37 μm)	Sandblasting and acid etching, Oxidation	micro‐structured SLA TiO_2_ surface (*R*_a_ =1.76 μm)	Screw Ø = 4.1 mm *L* = 10 mm Rabbit femoral condyles	Week 12	SLA surface showed superior roughness compared to the oxidized surface. However, similar BIC for both groups	Velasco‐Ortega et al.^82^
MAO‐treated machined TiO_2_ surface	Machining (MA) followed by Micro‐arc oxidization (MAO)	SLA Ti	Screws Ø = 3.3 mm *L* = 10 mm Rabbit femoral condyle	Week 4	MAO surface was superhydrophilic and showed slightly higher amount of bone formation compared to the SLA surface	Zhou et al.^83^
Ordered TiO_2_ nanotubes	Double acid etching and anodization	Microporous TiO_2_ surface	Disc/screw Ø = 10 mm Th = 3 mm Rat tibia	Week 2, 6	The nano‐tubular surface showed superior wettability, improved peri‐implant bone formation, and osseointegration	Pelegrine et al.^84^
Micro‐nano‐porous TiO_2_ structured surface (SLAffinity‐Ti) (*R*_a_ = 1.0 μm)	Grit‐blasting with Al_2_O_3_ particles, acid etching and electrochemical oxidation	Machined‐smooth TiO_2_ surface (*R*_a_ = 35 nm) Micro‐structured TiO_2_ rough surface (SLA) (*R*_a_ = 1.2 μm)	Screw Ø = 4 mm *L* = 8 mm Minipig tibia and mandible	Week 2, 4, 8	The nano‐porous structured surface (SLAffinity‐Ti) showed best biocompatibility with blood and improved osseointegration compared to the control surfaces	Ou et al.^86^
Nano‐tubular TiO_2_ surface	Grit blasting and double acid‐etching and electrochemical anodization	Machined‐smooth TiO_2_ surface	Flat implant Ø = 4 mm Th = 500 μm Mouse calvaria	Day 3, 7, 11, 15, 21, 28, 42	The nano‐tubular surface showed superior blood vessel density, BV/TV, and BIC compared to the machined surface	Khosravi et al.^85^
SLActive—moderately rough hydrophilic‐TiO_2_	SLA: Large‐grit sandblasting and double‐acid etching, SLActive: additional chemical treatment	SLA—moderately rough hydrophobic‐TiO_2_	Dome Ø = 5 mm *H* = 3 mm Rabbit calvaria	Day 4, 7, 14	Hydrophilic‐SLA group showed lower inflammatory response and increased osteogenic activity at early stage of healing	Calciolari et al.^41^, ^87^
Micro‐structured CaMg‐incorporating surface (*R*_a_ = 0.89 μm)	SLA and CaMg micro‐particle blasting	Micro‐structured surface (*R*_a_ = 0.76 μm)	Cylindrical screw Ø = 4 mm *L* = 8 mm Rabbit proximal tibia	Week 4, 6	Ca–Mg deposition increased osseointegration shown by enhanced BIC and bone mineralization level	Gehrke et al.^88^
Micro‐rough SLA surface modified with nanostructured strontium‐oxide layer (*R*_a_ = 2.35 μm)	SLA metallic‐oxide incorporation via hydrothermal treatment	Moderately rough SLA‐surface (*R*_a_ = 2.20 μm)	Screw Ø = 4 mm *L* = 8 mm Rabbit tibia and femoral condyle	Week 3, 6	The incorporation of strontium stimulated early bone formation and improved osseointegration as shown by higher BIC and removal torque	Fan et al.^89^
Na‐incorporated moderately rough hydrophilic TiO_2_ (*S*_a_ = 0.99 μm)	Sandblasting and acid etching and alkali treatment	Moderately rough hydrophobic‐TiO_2_ (*S*_a_ = 1.03 μm)	Screw Ø = 2.9 mm *L* = 10 mm Sheep tibia	Day 7, 14, 21, 28	The hydrophilic activated SLA surface showed superior BIC and bone area compared to the untreated‐SLA from day 14	Sartoretto et al.^90^
Grit‐blasted TiO_2_ Titania NT Titania NT loaded with Sr	Grit‐blasting, electrochemical anodization and heat treatment	Grit‐blasted surface	Screw Ø = 3 mm *L* = 6 mm Rat femur Cylindrical implant: Ø = 1 mm *L* = 12 mm Rat tibial condyles	Week 12	Titania NT loaded with Sr had the highest BIC among the tested groups	Dang et al.^91^
TiO_2_ blasted implant and Zoledronic acid treatment	Blasting, anodic oxidation and coating via immersion	TiO_2_ blasted implant	Screw Ø = 3,75 mm *L* = 7 mm Rabbit femoral condyle	Week 3	Inclusion of Zoledronic acid significantly improved implant stability, enhanced bone formation and osseointegration compared to control	Kwon et al.^92^
Anodized TiO_2_ (NanoTi) NanoTi + HAp deposition	Anodization and HAp deposition	Machined TiO_2_	Nail: Ø = 2 mm *H* = 20 mm *L* = 10 mm *B* = 3 mm *H* = 1 mm Rat femur	Week 10	Anodization and HA deposition improved osseointegration than control. NanoTi surface had comparable effect as NanoTi+HAp surface	Sirin et al.^57^
Hydrophilic, porous nano‐micrometer roughness (bimodal pores nm – 6 μm); Incorporation of Ca, P, O_2_	Anodization (electrolyte solution: glycerphosphate disodium salt, calcium acetate)	MA Ti MA TiZr anodized TiZr	Disc Ø = 10 mm, *H* = 1.5 mm Sheep femur	Week 4	Anodization lead to enhanced early osteointegration	Sharma et al.^93^
5% strontium (Sr) incorporated HAp‐coated TiO_2_ 10% Sr incorporated HAp‐coated TiO_2_ 20% Sr incorporated HAp‐coated TiO_2_	Polishing, acid‐etching and calcium chloride treatment, Coating via electrochemical deposition	HAp‐coated TiO_2_	Rod Ø = 1.2 mm *L* = 15 mm Ovariectomized rat distal femur metaphysis	Week 12	Incorporation of strontium into the HAp coating improved bone formation at the BIC. 20% Sr‐HAp surface showed the best osseointegration and mechanical strength	Tao et al.^94^
HAp‐coated (*R*_a_ = 2 μm) Grit blasted (*R*_a_ = 6 μm) Laser‐textured surfaces	Machining, Blasting, Coating via plasma spraying and Laser texturing	Machined (*R*_a_ = 0.1 μm)	Tapered pin Ø = 5–4 mm *L* = 3 mm Sheep tibia	Week 6	All modified implant surfaces revealed higher BIC relative to the machined surface. However, the BIC of the HAp‐coated surface was more superior than the blasted and laser‐textured surfaces	Coathup et al.^54^
UV‐treated SLA surface	Sandblasting using Al_2_O_3_, acid‐etching and UV treatment	Micro‐structured TiO_2_ rough surface (SLA)	Screw Ø = 3 mm *L* = 7 mm Rabbit tibia	Day 10, 28	UV treatment increased BIC and osseointegration	Lee et al.^95^
Hydrophilic microporous TiO_2_ microfiber (87% porosity)	Enfolded titanium microfibers, acid etching and UV treatment	Moderately rough TiO_2_ microfiber	Cylindrical implant Ø = 1 mm *L* = 2 mm Rat distal femur	Week 2, 4	Enhanced implant anchorage strength and bone formation at bone implant interface for UV treated implants	Park et al.^96^
HAp‐coated Ti surface (*R*_a_ = 90 μm) Bioactive glass coated Ti surface (*R*_a_ = 30 μm)	Coating via micro‐plasma spraying, and Vitreous enameling	MA TiO_2_ surface (*R*_a_ = 95 μm)	Cylindrical screw Ø = 3.5 or 4 mm *L* = 11 or 13 mm Human teeth (anterior maxilla and mandible regions)	1 year	The bioactive glass coated surface showed superior osteo‐integration in the maxillary region. Similar effect was seen in the mandibular region of the 3 groups	Mistry et al.^97^
CaTiO_3_ coating (pore size = 1–4 nm) HAp coating (pore size = 100–200 μm)	Coating via chemical (NaOH and CaCl_2_) treatment and plasma spraying	Uncoated MA TiO_2_ surface	Screw Ø = 2 mm *L* = 10 mm Rabbit femoral condyle	Week 2, 4, 8, 12	CaTiO_3_ and HAp coated surface showed comparable BIC and mechanical strength that was superior to the uncoated machined surface	Wang et al.^98^
Ca^+^ incorporated nano‐porous surface (*R*_a_ = 20.58 nm) Na^+^ incorporated nano‐porous surface (*R*_a_ = 21.46 nm)	Chemical (NaOH and CaCl_2_) and heat treatments	Machined surface (*R*_a_ = 70.25 nm)	Screw Ø = 1.2 mm *L* = 12 mm Rat femur	Week 1, 4, 8	BIC was higher in Na^+^ and Ca^+^ incorporated nano‐porous implants compared to the machined surface. Ca^+^ incorporation led to superior new bone formation in relation to the other groups	Su et al.^99^
Mg‐ion coated mesoporous TiO_2_ surface	Titania coating via spinning and heat treatment Metallic ion coating via physical deposition	Mesoporous TiO_2_ surface	Screw Ø = 1.5 mm *L* = 2.5 mm Osteoporotic rat tibia and femora	Day 1, 2, 7	The local release of Mg ion promoted rapid bone formation at the bone‐implant interface and the activation of osteogenic signals	Galli et al.^100^
Nanostructured Sr‐coating (Th = 1500 nm, with prewash in PBS) Nanostructured Sr‐coating (Th = 2000 nm, no washing) Nanostructured Sr‐coating (Th = 2000 nm, with industrial wash)	Coating via magnetron sputtering	Uncoated nanostructured surface	Rod Ø = 1.6 mm *L* = 5 mm Ovariectomized Rat tibia	Week 6, 12	At 6 weeks, Sr‐release significantly increased new bone formation and BIC. New bone formation was also higher at 12‐week but with no difference in the BIC compared to control. The best healing outcome was seen in design 2 which showed the highest Sr‐release content	Offermanns et al.^101^
10% polyphosphoric acid 1% Phosphorylated pullulan 10% phosphorylated pullulan 10% phosphorylated pullulan +1 μg BMP2	Coating via immersion	H_2_O‐treated surface	Screw with groove and thread Ø = 1.8 and 1.1 mm *L* = 3 and 1 mm Pig parietal bone	Week 4, 12	Ti‐implant surface functionalized with 10 wt% phosphate‐containing inorganic and organic polymers supported higher BIC and peri‐implant bone formation at earlier stage of bone healing	Cardoso et al.^102^
Graphene coated nanostructured surface	Chemical vapor deposition	Uncoated titanium	Cylindrical rods Ø = 5 mm *L* = 10 mm Rabbit femoral condyles	Week 4, 12, 24	Graphene nano‐coating enhanced osteogenesis and osteointegration via increased bone formation and mineralization with superior bone push‐out strength than the uncoated surface	Li et al.^103^
Pectin nanocoating (Rhamnogalacturonan‐I, RG‐I)	Implant surface amination (plasma polymerization of allylamine) followed by covalent coupling of RG‐I	Ti grade 2 without coating, Ti 2 aminated	Screw *L* = 8 mm Ø = 3.5 mm Rabbit tibia	Week 2, 4, 6, 8	Nanocoating with RG‐I showed no enhancement of osseointegration	Gurzawska et al.^104^
SLA‐Dopamine coating SLA‐Zoledronic acid coating SLA‐Dopamine +Zoledronic acid coating	Sandblasting and acid etching, Chemical coating via immersion	Micro‐roughened TiO_2_ (SLA)	Cylindrical implant Ø = 2 mm *L* = 4 mm Ovariectomized rat femur metaphysis	Week 8	Coating with Dopamine and Zoledronic acid sustainably improved osteointegration as revealed by the superior BIC and removal torque	Ma et al.^105^
Alkaline etched‐TiO_2_ with GL13K‐peptide coated surface	Alkaline etching, Peptide coating via silanization	Alkaline etched‐TiO_2_ surface	Screw Ø = 3.75 mm *L* = 7 mm rabbit femoral condyle	Week 6	Anti‐microbial GL13K‐peptide coated implant surface showed similar bone growth rate and osseointegration as the uncoated surface	Chen et al.^106^
Silicon‐substituted nano‐HAp coated surfaces (nano‐HAp‐Si)	Selective laser melting and precipitation coating	Porous Ti‐scaffolds	Disc Ø = 5 mm Rabbit femur	Month 2, 4, 6	Nano‐HAp‐Si coated scaffolds showed better osteointegration compared to the uncoated scaffolds	Ilea et al.^107^
Cell coating of smooth Ti (99.9% pure)	Enwrapping with cell sheet (MSCs, EPCs, or Co‐culture)	Smooth surface	Screw *L* = 6 mm Ø = 1.9 mm Rat tibia	Week 8	Cell sheet coated implants showed higher amount of mineralized bone and BIC compared to smooth implant. Co‐cultured cells gave the best results	Liu et al.^108^

### 5.1 Effect of micro–nano‐scale surface roughening of titanium implants

At the micrometer scale, moderately rough sandblasted and acid‐etched titanium surfaces inserted into the tibia of rabbits showed considerably higher RTVs at a later stage of the remodeling process. However, no difference between the modified and machined surface was observed regarding the BIC.^49^, ^74^

The combination of surface roughness at different length scales (micro, submicron and nanometer level), created by an overlay approach, prominently enhanced osteointegration, especially if the hybrid surface structures resembled the hierarchical architecture of natural bone.^49^, ^75^ The intermix of micro‐ and nano‐features increased the osteoconductivity at the implant interface, especially at the initial stage of the remodeling process. Using a combination of dual acid etching and nano‐texture blasting, Coelho and colleagues produced surfaces with nano‐to‐micrometer scale topographies and interestingly, the nano‐textured surface significantly improved the bone bonding strength after 9 days of implantation into a rat femur.^75^ A hierarchical micro‐to‐nano hybrid structuring can also be obtained by using site‐specific laser ablation and laser sintering methods.^49^, ^50^, ^76^, ^77^ Shah et al. and Trisi et al. showed an improved osteointegration of laser‐modified titanium implants characterized by a micro‐topography hybridized with a relatively thick nanostructured titanium‐dioxide layer.^50^, ^78^ This surface elicited a superior biomechanical anchorage at the bone‐implant interface in comparison to a just machined surface after 8 weeks of implantation in a rabbit metaphyseal tibia, as well as in a sheep iliac crest model. Using a rabbit femur implantation model, Cohen et al. reported augmented osteointegration of trabecular bone inspired porous titanium implants.^79^ These possessed a micro‐to‐nanoscale surface roughness, which was produced by a combination of grit‐blasting, acid etching and laser sintering. In comparison to a solid implant, this porous one showed a significantly higher new bone volume within and around the implant surface after 10 weeks of implantation. The porosity enabled direct bone ingrowth into the material pores, especially near the cortical bone interface. Chappuis et al. reported about the beneficial effects of nano‐patterning, leading to an increase in hydrophilicity and osteointegration surfaces in a miniature pig model compared to SLA, which was also true for compromised bone.^80^ Regarding the benefits of porosity, Zhang et al. already presented that a porous titanium substrate can achieve the same repair capacity as a porous HAp construct with titanium having the better biomechanical features.^81^

Another recent study comparing different surfaces produced via sand blasting / acid‐etching and oxidization, revealed a considerably higher micro‐roughness in the sandblasted and acid‐etched samples compared to the oxidized ones, which exhibited a lower roughness value *R*_a_.^82^ However, Zhou et al. reported that oxidized TiO_2_ implants presented a superhydrophilic surface properties with similar BIC and slightly higher bone formation compared to SLA.^83^

A combination of anodization with acid etching or blasting is a recently employed surface modification technique for generating submicron to nano‐textured hybrid implant surfaces for improved osteointegration.^84^, ^85^, ^86^ Indeed, when implanting these into rat tibia, mini‐pig tibia and mandible bones, there is better initial interactions with blood, superior BIC and improved biomechanical strength at the bone‐implant interface compared to machined control surfaces.^84^, ^86^ Similarly, titanium with nanostructured tubes on its surface implanted into a mouse calvaria defect model promoted neovascularization with fast maturation of the vasculature at the peri‐implant site. This resulted in early contact osteogenesis—the formation of bone directly on the implant surface—and faster osteointegration, facilitated by increased activities of local and remote osteogenic cells. ^85^

Micro‐rough topographies increase the surface area, thereby enabling stable and strong mechanical interlock between the implant and newly mineralized bone matrix, irrespective of the amount of new bone at the peri‐implant interface. Moderately rough surfaces at the micrometer scale facilitate higher mechanical stability/strength that promotes osteogenic differentiation. Generally, roughening of the surface is often achieved via the established SLA method. However, new methods, such as laser‐based techniques, can create more potent porous structures with better osteointegration, even if they exhibit lower roughness values than SLA.

### 5.2 Combination of surface roughening and chemical modification techniques

The conventional SLA method often yields a moderately rough and hydrophobic surface. The addition of chemical modification to SLA‐treatment improves the surface bioactivity, namely the surface wettability and energy, resulting in rapid contact with biological fluids and interaction with relevant biomolecules and osteogenic cells required to initiate the bone remodeling process. The improved early osseous healing response of a chemically activated hydrophilic SLA treated titanium surface (SLActive) was shown by Calciolari and co‐workers using a rabbit calvaria defect model.^87^ Proteomic analyses revealed that the hydrophilicity of the SLActive surface caused lower inflammatory response but increased the expression of prominent bone formation genes during the early stages of bone remodeling compared to normal SLA. Similarly, the deposition of bioactive substances onto the moderately rough micro‐structured surface can be achieved via blasting methods. Blasting Ca–Mg micro‐particles onto SLA surfaces produced moderately rough micro‐structured surfaces that were almost identical to the traditional SLA but differed in their biological response, which showed increased new bone formation with a superior microstructure.^88^ Incorporating Sr to a conventional SLA surface led to the generation of a novel bioactive SrO nanostructure layer with nano‐topographical features promoting early bone formation and ultimately enhanced osteointegration. BIC and RTV values were observed to be increased in comparison to conventional SLA implants after 6 weeks of inserting into the proximal tibia and femoral condyles of rabbits. ^89^ Similar to Sr, sodium modified SLA implants inserted into a sheep tibia revealed superior BIC during the early phases of osteointegration in comparison to untreated SLA‐implants.^90^

Other strategies to improve the bioactive capacity of Ti implants include the combination of anodization with chemical treatments. The anodization process leads to the formation of nano‐tubular structures (titania nanotubes) that permit higher and prolonged delivery of the incorporated chemical agents at the implant site. A study performed with the intercondylar notch of rat femora showed that an addition Sr^2+^ increased the bonding strength of titania nanotubes and promoted stronger bone‐implant interface interaction at 12 weeks postimplantation compared to the grit‐blasted control surface.^91^ This observation was corroborated by a study conducted with dental implants in a canine model, where the inclusion of Sr^2+^ stimulated osteoinduction and bone formation via promotion of angiogenesis and osteogenic signaling.^74^ Also nanotubes loaded with other compounds showed beneficial effects. For example, those with inserted zoledronic acid showed increased implant stability and RTVs after 3 weeks post in vivo implantation in the femoral condyle of rabbits compared to empty nanotubes.^92^ HAp loaded nanotubes inserted in rat femurs augmented the hydrophilicity and BIC when compared with blank nanotubes.^57^

Combining different chemical agents has also been reported to enhance bone formation. For example, Sharma et al. achieved earlier osteointegration of implants that were characterized by a hydrophilic, porous, nano to micrometer rough surface with an additional incorporation of Ca, P and O_2_ via anodization.^93^ Besides, the incorporation of Sr via electrochemical deposition into HAp coated Ti implants was able to substantially improve the quantity and mechanical strength of the newly formed trabeculae at the bone‐implant interface after 12 weeks of implantation into the femora of osteopenic rats.^94^

All these titanium implants surface modification techniques showed improved bone formation and osteointegration when compared to untreated surfaces. However, a comparison of multiple techniques, namely laser texturing, grit blasting and HAp coating, using an ovine model (large animal model in sheep) showed that these surface treatments resulted in similar roughness in the micron range but induced different effects on bone regeneration capabilities.^54^ The HAp treatment induced the highest BIC, but the laser structured surface attained similar values for interfacial strength and outperformed the other implants in RTV value and bone ingrowth, suggesting that it is the most preferable surface roughening method. In these studies, a surface harboring a more bone‐like composition, namely HAp coating, was inferior in the outcome compared to laser textured surface, despite their similar micro‐roughness. This indicates that the nano‐roughness of a surface is a highly significant factor for implant performance, which may be more significant than mimicking the bone micro‐scale. In particular for laser texturing, the created nano‐scale features appear in a foamy, roundly shaped morphology and have greater similarity to bone tissue, which is different to the SLA treated surfaces resembling rather sharp‐edged morphology (Figure 5).^51^ In accordance, Souza et al. concluded that proper nano‐texturing leads to a faster osteointegration process and furthermore, can reduce the risk of bacterial contamination.^27^

### 5.3 Effect of additional functionalization and bioactive coating

Further approaches that have been successfully applied to enhance the bioactive properties of a titanium implant surface are the functionalization or coating with specific molecules.

One method of functionalization is photo functionalization via ultraviolet light immediately prior to implantation. For example, this approach provoked an increased amount of bone mineralization and osteoblast proliferation at the early stages of healing compared to the standard SLA.^95^ Interestingly, the UV treatment in addition to increasing surface roughness, also led to the formation of superhydrophilic surface characteristics that promoted beneficial physicochemical changes and increased bone healing. Likewise, UV‐treated microfiber implants inserted into the rat femur promoted better implant anchorage and bone formation after 4 weeks compared to the non‐UV‐treated control group.^96^

Implantation of HAp and bioactive glass coated implants into human jawbones showed better biocompatibility with the surrounding tissue when compared to machined implants. These findings indicated that an improved surface hydrophilicity positively impacts the surface energy, thereby promoting the adhesion and proliferation of osteoblasts and relevant growth factors required for bone formation.^97^

Certain metallic ions such as calcium, magnesium, sodium and strontium have also demonstrated synergistic effects on osteogenesis. For example, the incorporation of calcium ions (Ca^2+^) into the titanium surface enabled the conversion of passive oxide into a bioactive oxide (CaTiO_3_), which is more favorable for biological interaction. Wang et al. reported excellent biocompatibility and osteointegration effects of nano‐bioactive CaTiO_3_ coated screws produced via treatment with NaOH and CaCl_2_.^98^ The results after 12 weeks of implantation were comparable to HAp‐coated and superior to uncoated implants.^98^ Ca^2+^ deposition in a nano‐porous Ti alloy equally resulted in improved osteoconductivity and overall bone formation at week four and eight after implantation in a rat femur compared to Na^+^ incorporation. The divalent Ca^2+^ incorporates deeper into the layer of the nano‐porous structure, enabling a consistent and sustained release over time, leading to a superior bioactivity and increased trabecular bone formation.^99^ Like calcium, magnesium is also vital in the process of bone regeneration, it promotes osteogenic differentiation, as well as angiogenesis. The integration or Mg^2+^ into Ti surfaces has led to an increased surface bioactivity and osteointegration. Interestingly, Mg released from mesoporous titanium films significantly supported bone formation after 7 days of implantation into the tibia and femora of osteoporotic rats. In addition, a positive osteogenic effect of Mg^2+^ doped surfaces compared to uncoated could be demonstrated by a 3‐fold higher expression of BMP6, a key growth factor involved in bone formation.^100^ Another important bioactive metal is Sr, which is known to enhance bone formation by stimulating osteoblastogenesis and inhibiting osteoclast formation. Ti surfaces with incorporated Sr^2+^ were shown to have beneficial effects on osteointegration, particularly based on the sustainable release over time. Using an osteoporotic rat tibia model, Offermans et al. demonstrated the bone regenerating effect of nanostructured Ti implants functionalized with different concentrations of Sr^2+^.^101^ After 6 and 12 weeks of implantation, these materials provoked significantly higher bone formation and osteointegration compared to the uncoated surface.

Besides surface coating with bioactive metallic ions, surface functionalization using organic and inorganic biopolymers has also been explored. For example, polyphosphoric acid and phosphorylated pullulan (a polysaccharide) have been demonstrated to facilitate early peri‐implant bone healing and osteointegration after 4 weeks of implantation into a porcine bone defect model.^102^ Moreover, using Graphene (2D modification of carbon with special nano‐topography and a characteristic rigid and rough structure) to coat a nanostructured Ti surface promoted osteointegration in a rabbit femur implantation model.^103^ However, not every functionalization leads to a significant improvement. A promising approach with pectin nanocoating by plasma polymerization could not yield detectable elevated osteointegration levels in comparison to the control surface.^104^

Coating of SLA‐treated titanium implants with the osteoinductive hormone molecules dopamine (involved in osteoblast differentiation and mineralization) and zoledronic acid (possesses a positive effect on new bone formation), for example, significantly enhanced implant integration, 8 weeks after implantation into the femur metaphysis of osteoporotic rats. In comparison to the SLA surface, dopamine and/or zoledronic acid coated implants showed a superior BIC rich in trabecular microstructure, that was further proven by significantly higher RTVs. Dopamine coating facilitated bone formation by inhibiting the expression of genes associated with osteoclast differentiation.^105^ Similarly, titanium implant surface coating with antimicrobial agents, such as the bactericidal cationic peptide GL13K, not only inhibited microbial activity but also promoted osteointegration after 6 weeks of implantation in a rabbit femur model.^106^ The addition of silicon‐substituted nano‐HAp to the surface of a selective laser structured titanium implant, inserted into the rabbit femur, promoted more organized bone formation, especially at the later stages of bone healing compared to implants without additional chemical treatment.^107^ Besides coating with biomolecules, compounds or ions, approaches with cell coating have arisen. The cells used, are those which are naturally available at the bone implant interface. Liu et al. showed improved osteointegration of titanium implants enwrapped with co‐cultured BMSCs and endothelial progenitor cells (EPCs) cell sheets after 8 weeks of implantation in irradiated rat tibia compared to machined‐smooth surfaces.^108^

Additional coating of structured titanium implants with bioactive materials is a surface modification technique for enhancing both the surface chemistry and topography in favor of pro‐osteogenic features. In vitro priming of implant surfaces with living cells that are present in bone tissue can be the next step of surface functionalization further mimicking the native bone environment. Due to more elaborate ethical and preparatory processes prior to implantation, this approach will require a lot more investigation before application in clinical daily life.

To summarize this chapter, the key factors in all reviewed experiments were the optimization of coating techniques and the combinations with structuring methods to ensure the optimal contribution of various bioactive agents to osteointegration improvement. The combination of chemical treatment with other surface topography modification techniques has led to the development of novel titanium‐based implant surfaces with improved micro‐to‐nano hybrid topographies. Their enhanced bioactive properties facilitate earlier bone regeneration and could lead to improved osteointegration at the bone‐implant interface in both healthy and compromised bone.

## 6 CONCLUSION

The current research on the osteointegration capacity of titanium implants reports promising enhancement strategies via increasing porosity, hydrophilicity and nano‐structuring of the surface, frequently using a combination of roughening techniques and bioactive substance coatings.

In general, hydrophilic surfaces show improved osteoinduction and decreased inflammatory response, and when combined with nano‐patterning, augmented osteointegration can be achieved. HAp, the primary inorganic component of bone tissues, has been investigated as a coating material for a long time and is still frequently chosen. Its deposition can promote better BIC, as well as bone tissue formation and is already in clinical use for cementless fixated implants. However, new coating compositions, such as calcium titanate or bioactive glass, arise as promising candidates for implant surface modification. In sum, creating a rough, nano‐textured surface and sequential application of various techniques to further biofunctionalize the implant is desired. Next to coating with bioactive molecules, another interesting approach is surface loading with cells. This type of functionalization has not been vastly studied, as its clinical translation is more challenging due to the cell preparatory requirements and regulations. Still, this approach can potentially gain more attention in the future, alongside the progression of cell‐based therapy and personalized medicine in many other clinical areas.

Nano‐structuring of titanium surfaces (e.g., via laser texturing), is a very attractive and expanding area, which should be further explored in great detail, as it holds the potential to induce high osteointegration and biomechanical anchorage without additional coatings. Micro‐ and nano‐porous titanium substrates are able to achieve the same repair capacity as porous HAp constructs, with titanium having more suitable biomechanical features, suggesting that the surface nanostructure is of great importance for proper bone formation. Hence, in the future, even more attention will be paid not only to the micro‐scale modifications, but also to the nano‐patterning of novel implants for augmented osteointegration.

In the process of developing next‐generation‐implants, it will be of great importance on behalf of the biological assessment, as well as cross‐study comparability, to improve certain evaluation parameters. These parameters include the use of primary human cells in addition to cell lines, analyzing cell responses at both mRNA and protein levels, performing cell monitoring over longer periods of time in vitro and in vivo, and carrying out precise histomorphometry of the tissue at the implant interface.

All in all, metal implants for bone and joint repair have demonstrated a tremendous success in the last decades. Nevertheless, new methodologies for surface modifications via laser texturing, and for boosting material antibacterial properties via novel coatings, can specifically target clinical needs, such as reduction of implant loosing and infection risk. Gaining in‐depth knowledge on bone cell–implant interactions can be implemented to further unleash the potential of emerging technologies to create designer implants targeting different patient cohorts.

## CONFLICT OF INTEREST

The authors have no conflicts of interest to declare.

## AUTHOR CONTRIBUTIONS

**Theresia Stich:** Conceptualization; investigation; visualization; writing‐original draft; writing‐review & editing. **Francisca Alagboso:** Conceptualization; investigation; visualization; writing‐original draft. **Tomáš Křenek:** Funding acquisition; writing‐review & editing. **Tomáš Kovářík:** Funding acquisition; writing‐review & editing. **Volker Alt:** Funding acquisition; validation. **Denitsa Docheva:** Conceptualization; funding acquisition; project administration; supervision; validation; writing‐review & editing.

7

### PEER REVIEW

The peer review history for this article is available at https://publons.com/publon/10.1002/btm2.10239.
